# Promotion effect of angelica sinensis extract on angiogenesis of chicken preovulatory follicles in vitro

**DOI:** 10.1016/j.psj.2022.101938

**Published:** 2022-04-30

**Authors:** Hao Chen, Xin Chen, Zhenlei Ping, Xiaowen Jiang, Ming Ge, Jun Ma, Wenhui Yu

**Affiliations:** ⁎ College of Veterinary Medicine, Northeast Agricultural University, Harbin 150030, PR China; † Institution of Traditional Chinese Veterinary Medicine, Northeast Agricultural University, Harbin 150030, PR China; ‡ Key Laboratory of the Provincial Education Department of Heilongjiang for Common Animal Disease Prevention and Treatment, Harbin 150030, PR China

**Keywords:** *Angelica sinensis* extract*,* preovulatory follicles, angiogenesis, follicle microvascular endothelial-like cell, granulosa cell

## Abstract

Preovulatory follicles need a network of blood vessels to growth and maturation in hens (*Gallus gallus*). *Angelica sinensis* (Oliv.) (**AS**), a traditional Chinese herb, displays a novel pro-angiogenic activity. The molecular mechanisms underlying AS promoting preovulatory follicles angiogenesis are poorly understand. Several recent studies investigated the expression of vascular endothelial growth factor A (**VEGF-A**) in angiogenesis. In order to explore the promotion effect of AS extract on angiogenesis of chicken preovulatory follicles, we studied the effect of AS extract on follicle microvascular endothelial-like cells of chicken (**FMEC**) and granulosa cells (**GC**). The current study indicated that AS extract could promote the proliferation of FMECs and GCs. The assays of wounding healing, transwell invasion and tube formation showed that AS extract could enhance the invasion and migration ability of FMECs in vitro. The results of western blot and RT-PCR showed that AS extract promoted the phosphorylation of vascular endothelial growth factor receptor 2 (**VEGFR2**) in FMECs by activating the PI3K/AKT signaling pathway. The AS extract activated PI3K/AKT signaling pathway and up-regulated the expressions of hypoxia-inducible factor 1-α (**HIF1-α**) and VEGF-A in GCs. In addition, treatment of FMECs and GCs with LY294002 (a PI3K inhibitor) significantly down-regulated the phosphorylation of VEGFR2, VEGF-A, and HIF1-α. The mRNA expression levels of PI3K, AKT, VEGF-A, VEGFR2, and HIF1-α were consistent with protein expression levels. In conclusion, our research showed that AS extract can promote the follicle angiogenesis in hens in vitro, providing a basis for application of the traditional Chinese herb AS in poultry production.

## INTRODUCTION

Efficient blood circulation is the prerequisite for follicle development in laying hens (Ma et al., 2020). Within the fully developed ovary of hens, the percentage of blood flow is greatest to the 5 largest preovulatory follicles and significantly increases with increased follicle size and at the time of ovulation (Scanes et al., 1982). After selection, the development of preovulatory follicles requires a large amount of nutrients, rapidly growing from 9 mm to more than 40 mm, which requires a strong vascular network to deliver nutrients to preovulatory follicles (Johnson and Woods., 2009). As the follicle develops, the theca layer becomes increasingly more vascularized except for the stigma. The largest arteries from the follicle stalk are directed toward the fastest growing follicles, branch into arterioles, and pass through the theca to the basal lamina to form arterial capillaries (Gilbert, 1971). The granulosa layer and theca layer in hens' preovulatory follicles remain independent, which is similar to the formation of the mammalian corpus luteum (Rico et al., 2014). In primates, angiogenesis of corpus luteum through the traditional mechanism of growth from the existing thecal vasculature to the granulosa layer, but the granulosa layer is always devoid of angiogenesis. It means that granulosa layer induces vascular endothelial cells to proliferate, and makes vascular endothelial cells migrate toward the granulosa layer. (Kaessmeyer and Plendl, 2009). The developed preovulatory follicle is a highly vascularized tissue with microvascular endothelial cells accounting for 50% of the total cell count (Kim et al., 2016).

*Angelica sinensis* (Oliv.) Diels (**AS**) is a member of the Umbelliferae family. AS, also called Dong Quai, is a traditional medicine commonly used to treat diseases in China and as a food supplement in Europe and North America (Hsu et al., 2014). The efficacy of AS involves in promoting blood circulation, pain relief, anti-inflammatory, antioxidant, and treatment for anemia and cardiovascular (Han et al., 2021). It is also used as a medicinal food in meat and soup, and is often used with other medicinal herbs (Hook, 2014). The rhizome of AS contains more than 70 compounds, most of which have been isolated and identified, including polysaccharides, organic acids, and phthalides (Jin et al., 2012; Yeh et al., 2012; Ma et al., 2015). Ferulic acid and caffeic acid account for a relatively high proportion in AS, which is proved to augment angiogenesis (Lin et al., 2010; Tu et al., 2020). Early studies have shown that AS extract could promote the proliferation of human umbilical vein endothelial cells (**HUVECs**) and mouse heart microvascular endothelial cells, and increase the number of CAM vessels in chicken embryo (Zheng et al., 2006; Meng et al., 2008).

Angiogenesis, the formation of new blood vessels from existing ones, involves a series of molecular biological changes (Robinson et al., 2009). Many pro-angiogenic factors have been identified, although angiogenesis is a highly complex process. The main studies on the promotion of angiogenesis focus on the secretion of VEGF-A, binding of VEGF-A to VEGFR2 on endothelial cells and the expression of HIF1-α in tissue cells (Bowler and Oltean, 2019; You et al., 2021). During the development in preovulatory follicles, granulosa cells secrete a large number of cytokines, especially HIF1-α, which can be transferred to the nucleus and regulate the secretion of VEGF-A, further promote angiogenesis in theca layer (Bahramrezaie et al., 2019). PI3K and AKT are key downstream proteins of VEGFR2 and upstream proteins of the HIF1-α/VEGF-A signaling pathway (Zhang et al., 2020; Yang et al., 2021).

In this study, the effects of AS extract on the angiogenesis of follicle microvascular endothelial-like cells of chicken (**FMEC**) in vitro were verified by wound healing, migration and tube formation experiments. Western blot was used to detect the effects of AS extract on the protein expression of VEGFR2, PI3K, and AKT in FMECs and PI3K, AKT, HIF1-α, and VEGF-A in granulosa cells (**GC**), providing theoretical basis for developing feed additives to maintain a high productivity of laying hens and improve eggs quality.

## MATERIALS AND METHODS

### Ethics Statement

Animal care in this study was performed in accordance with the Animal Experiment Management Regulations (Ministry of Science and Technology of China, 2004) approved by the Animal Care and Use Committee of Northeast Agricultural University, China.

### Preparation of Aqueous Extract of AS

The air-dried herbs used in this study were purchased from a Chinese herbal medicine company (Gansu, China), identified by professors from institute of Chinese Veterinary Medicine, Northeast Agricultural University, and stored at 4°C. Took 100 g dried AS, added 1,000 mL distilled water, and soaked for 2 h at room temperature. The solution was heated to 100°C and remained for 2h. Then the solution was filtered to remove insoluble materials and freeze-dried at −50°C. The average yield of AS was 22.3% (W/W). The freeze-dried was stored at −20°C, dissolved in DMEM and filtered (0.22 μm) for all experiments (Meng et al., 2008).

### LC-MS

The LC analysis was performed on a Vanquish UHPLC System (Thermo Fisher Scientific, Waltham, MA). Chromatography was carried out with an ACQUITY UPLC HSS T3 (150 × 2.1 mm, 1.8 μm; Waters, Milford, MA). The column maintained at 40°C. The flow rate and injection volume were set at 0.25 mL/min and 2 μL, respectively. For LC-ESI (+)-MS analysis, the mobile phases consisted of (C) 0.1% formic acid in acetonitrile (v/v) and (D) 0.1% formic acid in water (v/v). Separation was conducted under the following gradient: 0–1 min, 2% C; 1–9 min, 2–50% C; 9–12 min, 50–98% C; 12–13.5 min, 98% C; 13.5–14 min, 98–2% C; 14–20 min, 2% C. For LC-ESI (-)-MS analysis, the analytes was carried out with (A) acetonitrile and (B) ammonium formate (5 mM). Separation was conducted under the following gradient: 0–1 min, 2%A; 1–9 min, 2–50%A; 9–12 min, 50–98%A; 12–13.5 min, 98%A; 13.5–14 min, 98–2%A; 14–17 min, 2%A.

Mass spectrometric detection of metabolites was performed on Q Exactive Focus (Thermo Fisher Scientific) with ESI ion source. Simultaneous MS1 and MS/MS (Full MS-ddMS2 mode, data-dependent MS/MS) acquisition was used. The parameters were as follows: sheath gas pressure, 30 arb; aux gas flow, 10 arb; spray voltage, 3.50 kV and −2.50 kV for ESI(+) and ESI(-), respectively; capillary temperature, 325°C; MS1 range, m/z 81–1,000; MS1 resolving power, 70000 FWHM; number of data dependant scans per cycle, 3; MS/MS resolving power, 17500 FWHM; normalized collision energy, 30%; dynamic exclusion time, automatic.

### Isolation and Culture of GCs

Ten healthy Hy-line White laying hens aged 30 wk which laying rate was more than 90% were used in this study. One hen was euthanized for each repeated experiment. Hens were euthanized and immersed in 75% alcohol for 5 min, preovulatory follicles (F1–F5 follicles) were moved out. Preovulatory follicles (F1–F5 follicles) were immediately placed in pre-cooled PBS buffer containing 1% penicillin-streptomycin (Solarbio, Beijing, China) to wash blood stains for 3 times. A scalpel was used to make a quick incision in the follicle, and the follicle was quickly inverted to release the yolk. The granulosa cell layer along with the yolk was discharged into the pre-cooled PBS. The theca layers were placed in the pre-cooled DPBS for later use. The granulosa cell layers were put into 1 mg/ mL type II collagen (Solarbio), repeatedly blown and mixed, and digested in 37°C constant temperature water bath for 8 min. And then, M199 medium (HyClone, Logan, UT) was supplemented with 10% FBS (Clark Bioscience, Virginia, USA) and 1% penicillin-streptomycin was added, filtered by nylon mesh (70 μm), and centrifuged at 250 g for 5 min (Hao et al., 2021). The pellet was resuspended in M199 medium (containing 10%FBS and 1% penicillin-streptomycin) and the cells were cultured at 37°C under 5% CO_2._

### Isolation and Culture of FMECs

After the excess connective tissue and blood stains removed from the theca lays, the theca layers were digested in DPBS containing 1 mg/ mL type I collagenase (Solarbio), 0.4mg/ mL DNase (Solarbio) and 0.1%BSA for 60 min in a constant temperature water bath at 37°C. DMEM (HyClone) (containing 10% FBS) was added, filtered by nylon mesh (70 μm) and centrifuged at 250 *g* for 5 min. Meanwhile, pre-cooled 35% Percoll (Solarbio) was centrifuged at 30,000 *g* at 4°C for 15 min. The cell pellets were resuspended in DMEM and tiled on the centrifuged Percoll. After centrifuging for 1 min at 400 *g* in a vertical centrifuge, the solution was divided into 3 layers. Cells in middle layer (a density of 1.033–1.047g/ Ml; Hou et al., 2020) were carefully absorbed into a 15 mL centrifuge tube, and centrifuged for 5 min at 250 *g*. The supernatant was discarded. The cell pellets were resuspended by ECM medium containing 10%FBS, 50 μg/mL ECGs (Sigma-Aldrich, St. Louis, MO), and 1% penicillin-streptomycin double antibody. The cells were cultured at 37°C under 5% CO_2_ for 24 h, changing the ECM medium every 2 to 3 d. Only 3 to 6th generations of cells were used for follow-up tests (Sakurai et al., 2011).

### Cell Activity Assay

Cell viability was measured by cck-8 assay. FMECs and GCs cells were cultured in 96-well plates treated with different concentrations (25, 50, 100, 200, 400 μg/mL) of AS for 24 h. Then, liquid in the plates was changed into complete medium without AS. Cck-8 (Solarbio) was added to each well and incubated for 2 h at 37°C. The absorbance at 450 nm was measured by full wavelength microplate reader (Bio Tek, Vermont, USA).

### Wound Healing Assay

The 5 × 10^5^ cells/mL FMECs were cultured in the 6-well plate until the cells full confluence and began to scratch. After using a sterile pipette tip scratch, cell debris were removed with PBS buffer. The medium was added with or without LY294002 (10 μmol/L) 1 h prior to different AS concentrations (100 and 200 μg/ mL). After cultured in 37°C for 12 h, cells were observed and photoed under a microscope (Nikon, Tokyo, Japan).

### Transwell Invasion Assay

Spread a layer of Matrigel (Corning, Bedford, MA) on the 24-well cell culture chamber with 8-μm pore size polycarbonate filters (Corning). ECG medium was added into the lower compartment. The FMECs (2 × 10^5^ cells/mL) were cultured in the medium with or without LY294002 (10 μmol/L) for 1 h in advance and treated with different concentrations of AS extract (100 and 200 μg/mL) for 24 h. The lower membranes were fixed with 4% paraformaldehyde and stained by 0.1% crystal violet. Finally, a microscope was used to take pictures and analyzed by Image J.

### Tube Formation Assay

The FMECs (2 × 10^5^ cells/mL) treated by different concentrations of AS extract (100 and 200 μg/mL) with or without LY294002 (10 μmol/L) were seeded in plates for 6 h at 37°C which were pretreated with Matrigel. The tube formation state was observed by a microscope and analyzed by Image J.

### Enzyme Linked Immunosorbent Assay

The GCs (5 × 10^5^ cells/mL) treated with different concentrations of AS extract (100 and 200 μg/mL) with or without LY294002 (10 μmol/L) were seeded in plates for 48 h at 37°C. Cell media were harvested in sterile tubes and centrifuged (12,000 rpm, 10 min, 4°C) to obtain the supernatants. VEGF ELISA kit was purchased from Nanjing Jiancheng Bioengineering Institute (Jiancheng, Nanjing, China). The target protein concentrations were detected by the method consistent with the manufacturer's instructions.

### Real-Time-PCR

To measure the quantification of gene expression levels, quantitative real-time PCR (**QRTPCR**) was performed. FMECs and GCs were treated with and without LY294002 for 1 h, and then treated with different concentrations of AS extract for 48 h. Total RNA from cells was extracted using the TRIzol reagent (Thermo Fisher Scientific). The RNA samples were reverse transcribed to cDNA using a PrimeScript RT reagent kit (Thermo Fisher Scientific). QRTPCR was performed with SYBR Premix Plus (Tiangen Biotech CO. Ltd., Beijing, China). The primers for the various genes were listed in Table 1. Relative mRNA expressions were determined by 2^−△△Ct^ method.

**Table 1:** Primers used for real-time RT-PCR analysis.

Gene name	Forward (5’-3’)	Reverse (3’-5’)	Accession No.
PI3K1	TGACGCAGAGAGTGAGCAACAAG	GGATTGGAGGAGCAACATCAGGAG	NM_205518.2
AKT1	ACACAGAATCACCCACTTTCACCAC	CTGGATAACTCGCTCGTTCAGATGG	NM_001396387.1
VEGFR2	TGTGGTCATTTGGAGTTCTGCTGTG	TGGTGCTCTCATTCTTGTGCCTTC	NM_001004368.2
VEGF-A	CCTGTGTGCCTCTGATGAGATGTG	CGCTATGTGCTGACTCTGATGGG	AY168004.1
HIF1α	GAAGTCAAGAGATGCAGCCAGGTG	GGTCAGCCTCATAATGGATGCCTTG	NM_001396327.1
β-actin	CCAGCCATGTATGTAGCCATCCAG	GGTAACACCATCACCAGAGTCCATC	NM_205518.2

### Western Blot

FMECs and GCs were treated with and without LY294002 for 1 h, and then treated with different concentrations of AS extract for 48 h. Cells were lysed in RIPA lysis buffer to extract total proteins. The content of proteins was determined using BCA kit. The proteins were resolved on SDS-PAGE of gradient concentration and transferred to polyvinylidene difluoride membranes. After blocking, membranes were incubated with primary antibodies for the detection of CD31 (1:1,000, ABclonal, Wuhan, China; A0378), PI3K (1:1,000, ABclonal; A11402), P-PI3K (1:500, ABclonal; A4992), AKT (1:1,000, ABclonal; A18120), P-AKT (1:500, ABclonal; AP1068), VEGF-A (1:200, ABclonal; A12303), VEGFR (1:500, ABclonal; A11127), P-VEGFR (1:500, ABclonal; AP0382), HIF1-α (1:1,000, ABclonal; A0378), β-actin (1:2,000, ABclonal; AC038), and secondary antibodies. The epitope region used for creating the antibodies used is 90% homologous between rabbit and chicken according to protein blast. Protein bands were visualized with chemiluminescent system (Tanon, Shanghai, China) and quantified by Image J software.

### Immunofluorescence

Cells were fixed with 4% paraformaldehyde, permeabilized with 0.2% triton X-100 (Beyotime, Shanghai, China). After blocking with FBS, cells were incubated with primary antibodies CD31 (1:100, ABclonal; A0378), vWF (1:100, ABclonal; A1335) and DAPI (blue) (Beyotime) and Alexa Fluor594 (red) (1:200, Beyotime) conjugated second antibodies. Images were acquired using fluorescence microscopy (Nikon, Tokyo, Japan).

### Statistical Analysis

The data were analyzed and visualized using GraphPad Prism (version 8, San Diego, CA). The values were presented as mean ± S.E.M. Comparisons among the groups were evaluated using one way ANOVA with Tukey test. *P* value < 0.05 was considered statistically significance of difference.

## RESULTS

### LC-MS

**Figure 1:**
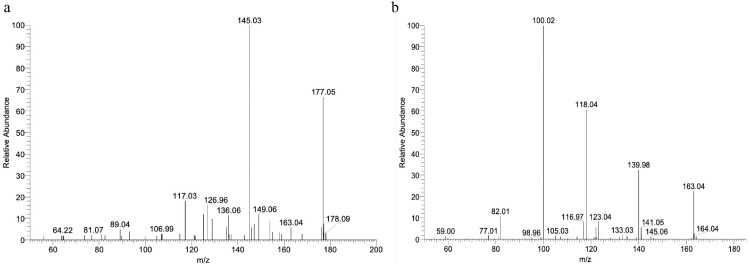
M/Z ratio obtained by LC-MS analysis. (A) Ferulic acid, (B) Caffeic acid.

The LC-MS analyses of AS extract were show in Figure 1, in which ferulic acid (m/z = 177.05; retention time = 8.90 min), caffeic acid (m/z = 163.03; retention time = 12.17 min), indicating the presence of ferulic acid and caffeic acid in the AS extract. The AS extract sample used in this study contained the active ingredients described in previous reports (Zhao et al., 2018).

### Characterization of Isolated FMECs

**Figure 2:**
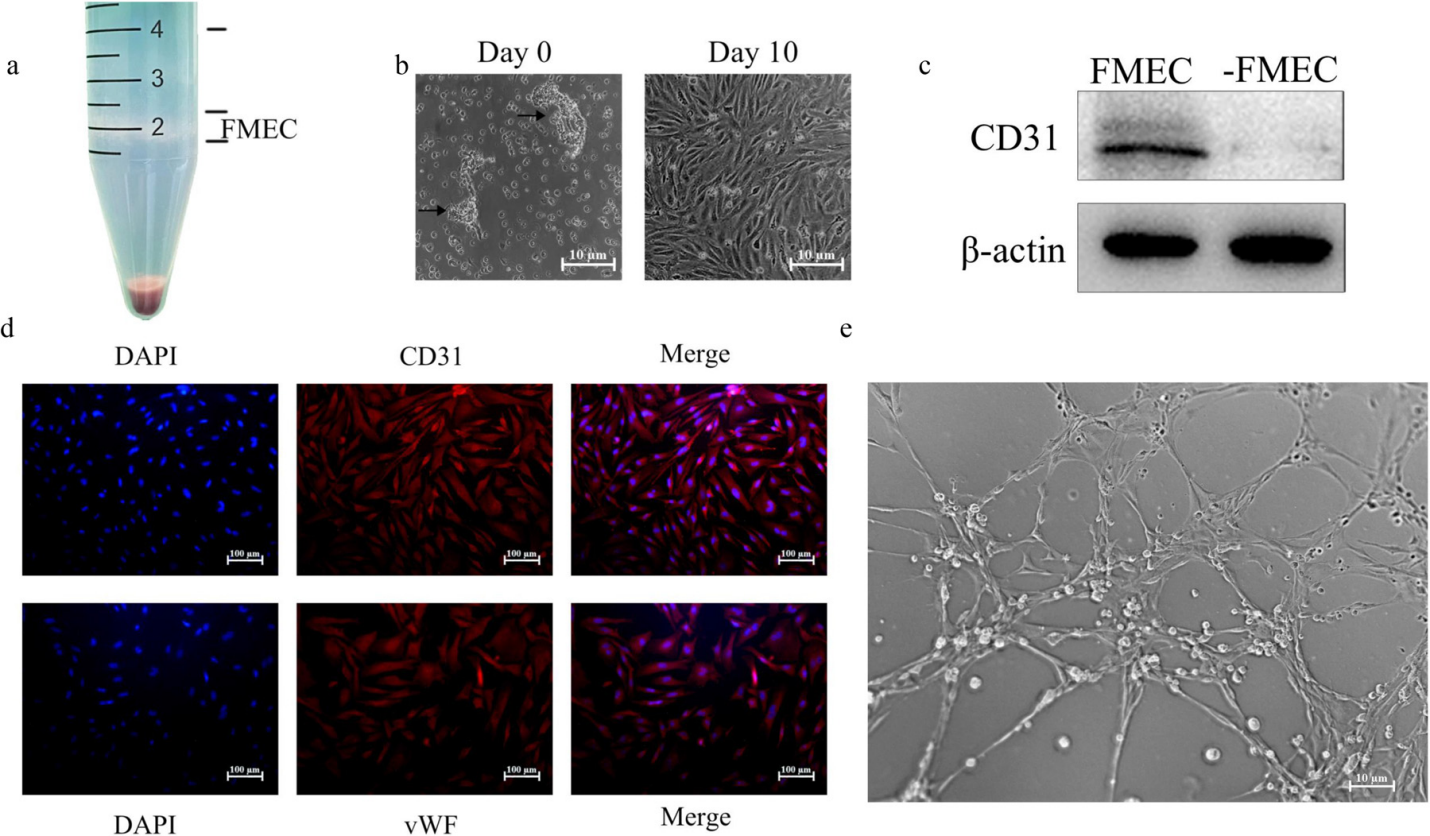
The characterization of isolated follicle microvascular endothelial-like cells of chicken (FMECs). (A) Theca layer dispersing cell centrifugation process, using density discontinuous Percoll solution to enrich microvascular endothelial cells. (B) FMECs were shown using phase-contrast microscopy (200 ×). (C) Western blot analysis of CD31 was performed on FMECs and -FMECs lysates containing 20 μg protein. (D) Immunofluorescence of FMECs, CD31 and vWF were coupled with Alexa Fluor 594 (red), and the nuclei were coupled with DAPI (blue) (200 ×). (E) FMECs were seeded on Matrigel for 6 h, representative photographs were shown (200 ×). Experiments (C-E) were performed in triplicate.

FMECs were isolated by discontinuous Percoll density gradient centrifugation method. The density of FMECs is 1.033 to 1.047g/ mL (Figure 2A). The isolated microvascular segments and FMECs were shown in Figure 2B. FMECs were single-layer adherent cells in spindle and polygon shape. FMECs and remaining cells in centrifuge tube (-FMEC) were detected by western blotting method, and CD31 was significantly expressed in FMEC, while almost not in -FMEC (Figure 2C). The isolated cells showed positive results of CD31 and von Willebrand Factor (**vWF**) detected by immunofluorescence. The cells were counted in 3 fields and repeated 3 times. The percentages of CD31 and vWF positive cells were (95.36% ± 6.58) % and (94.87 ± 5.32) % (Figure 2D). FMECs seeded on plates presented the formation of tubular structures which is a characteristic of microvascular endothelial cells (Figure 2E). Based on the above results, we believed that the isolated cells were microvascular endothelial cells of chicken follicles with high purity.

### Effects of AS Extract on the Viability of FMECs and GCs

**Figure 3:**
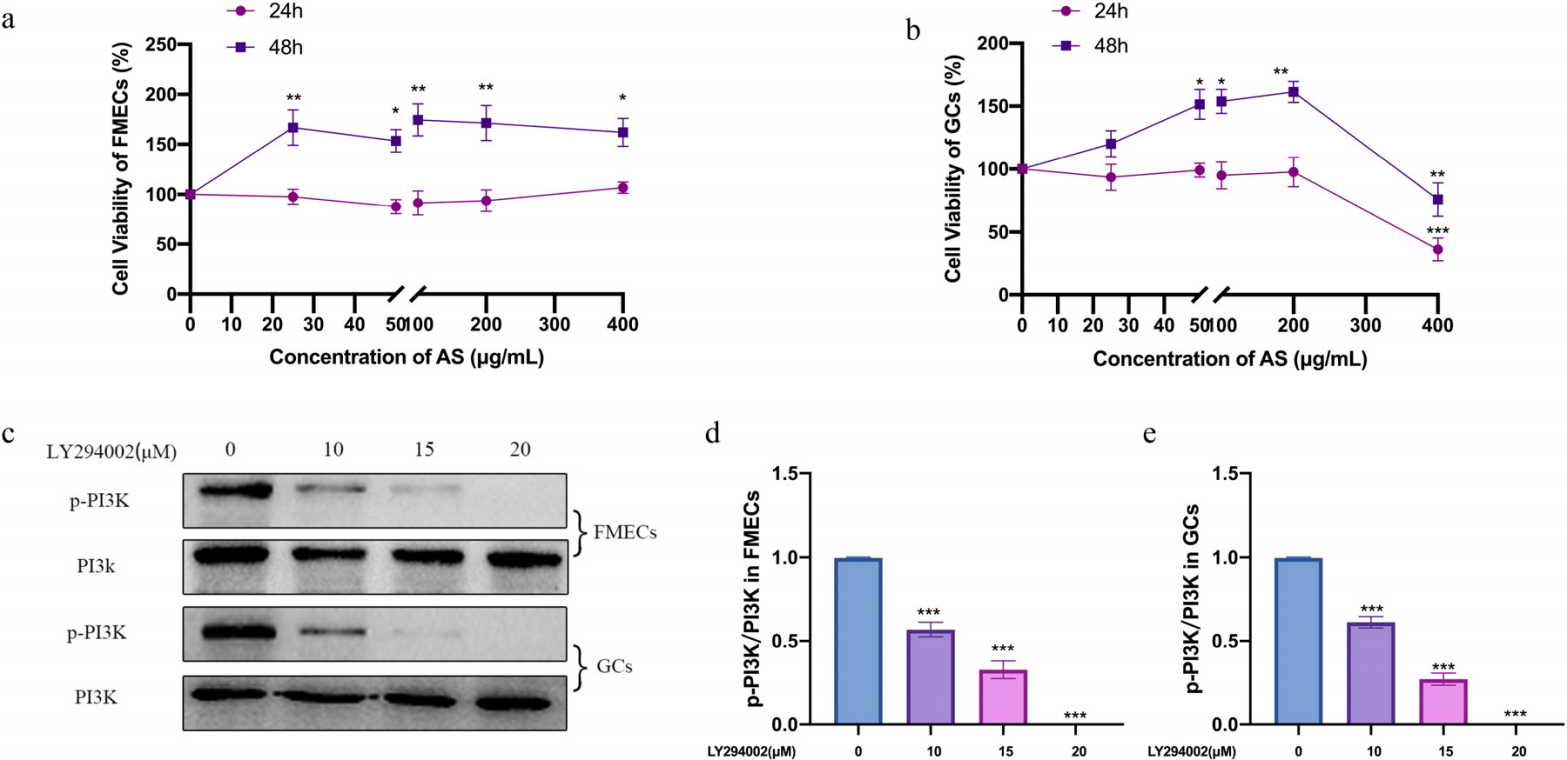
*Angelica sinensis* (AS) extract promoted the cell proliferation. Effects of LY294002 (a PI3K inhibitor) at different concentrations on follicle microvascular endothelial-like cells of chicken (FMECs) and granulosa cells (GCs). (A, B) FMECs and GCs were treated with AS (0–400μg/ mL) for 24 h and 48 h respectively. Cell proliferation was detected by CCK8 method. (C-E) Effects of LY294002 at different concentrations on p-P13K and quantitative analysis of p-PI3K/PI3K protein. All experiments were performed in triplicate, and the data are the mean ± S.E.M (*P < 0.05, **P < 0.01, ***P < 0.001).

Cck-8 was used to detect the effect of AS extract on the proliferation of FMECs and GCs. Compared with the control group, treating with different concentrations of AS extract for 24 h did not have any effect on the proliferation of FMECs and GCs (Figures 3A and 3B). However, after treating with AS extract for 48 h, the cell viability of FMECs significantly increased (*P* < 0.05) at each concentration compared with the control group. The cell viability of GCs significantly increased at 25, 50, 100, and 200 μg/ mL (*P* < 0.05, *P* < 0.01), and significantly decreased at 400 μg/ mL (*P* < 0.01). Both of FMECs and GCs proliferated most at 100 and 200 μg/ mL concentrations of AS. One hundred and 200 μg/ mL concentrations of AS were used in the subsequent experiments.

### Effects of LY294002 at Different Concentrations in FMECs and GCs

To assess the underlying mechanism of AS extract in FMECs and GCs, PI3K/AKT signaling pathway was detected. As shown in Figures 3C–3E, LY294002 administration decreased p-PI3K levels in a dose-dependent manner (10, 15, and 20 μM) in FMECs and GCs (*P* < 0.001). In this present study, we used LY294002 (15 μM) at the optimal concentrations.

### Effects of AS Extract on the Invasion and Migration Ability of FMECs

**Figure 4:**
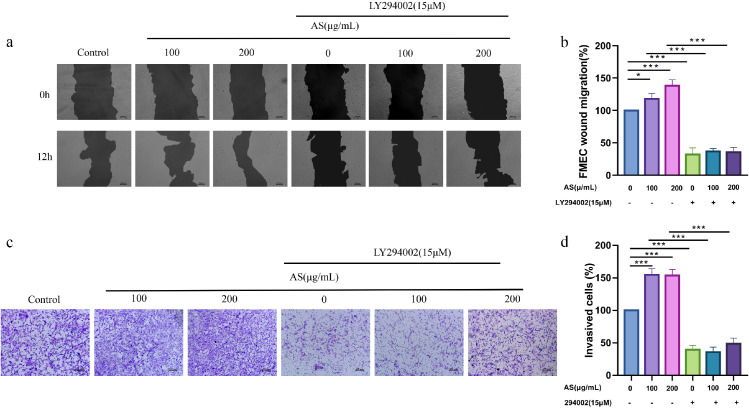
Effects of *Angelica sinensis* (AS) on migration and invasion of follicle microvascular endothelial-like cells of chicken (FMECs). (A, B) Cells migration ability of different groups were measured by wound healing (200 ×). Photographs from 0h to 12 h of the scratch were taken to measure the percentage of cell migration (200 ×). (C, D) FMECs treated with AS were seeded in Transwell chamber, ECG medium was added in the lower chamber, and the number of cells passing through Transwell chamber was calculated after incubation for 24 h. All experiments were performed in triplicate, and the data are the mean ± S.E.M (*P < 0.05, **P < 0.01, ***P < 0.001).

This study used wound healing assay in order to determine the effect of AS extract on FMECs migration (Figures 4A and 4B). Compared with the control group, the migration ability of cells treating with AS extract significantly enhanced (*P* < 0.05, *P* < 0.001) in a concentration-dependent manner. The migration ability of cells significantly decreased when treated with LY294002 alone, compared with the control group (*P* < 0.001), indicating that LY294002 could inhibit the migration ability of FMECs. When AS extract and LY294002 were added simultaneously, the migration ability of FMECs did not significantly improve as well.

The ability of invasion is also a measure of angiogenesis. Transwell invasion assays were used to detect invasion of FMECs (Figures 4C and 4D). Compared with the control group, the invasion ability of FMECs treating with AS extract significantly enhanced (*P* < 0.001). The invasion ability of FMECs significantly decreased when treated with LY294002 alone, compared with the control group (*P* < 0.001). When AS extract and LY294002 were added simultaneously, the invasion ability of FMECs did not significantly improve compared with that of LY294002 alone.

### Effects of AS Extract on the Tube Forming Ability of FMECs

**Figure 5:**
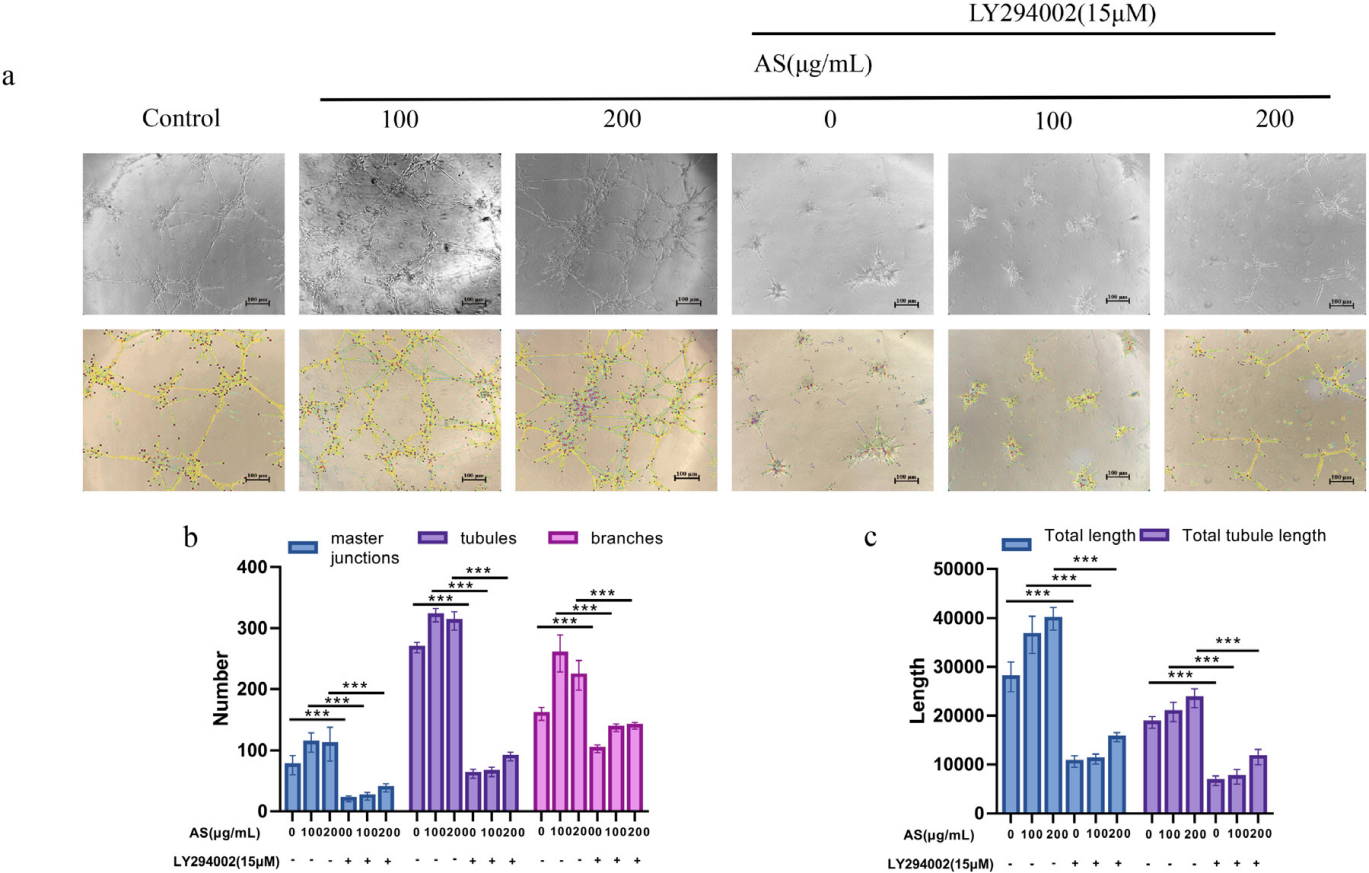
Effect of *Angelica sinensis* (AS) extract on tube form ability of follicle microvascular endothelial-like cells of chicken (FMECs). (A) Photographs of capillary-like structures in different groups of cells after the addition of AS were shown. (B,C) Image J software is used to measure the capillary network. Histograms, respectively, on behalf of the number of master junctions, tubules, branches, total length and total tubule length. All experiments were performed in triplicate, and the data are the mean ± S.E.M (*P < 0.05, **P < 0.01, ***P < 0.001).

Tube forming experiment is an important model of vascular formation in vitro. In this study, Matrigel was used to simulate the environment of vascular formation in vivo (Figures 5A–5C). The number of master junctions, tubules, branches, total length, and total tubule length in FMECs after AS extract processing increased significantly (*P* < 0.001), compared with the control group, and a better capillary network was observed. After the addition of LY294002 alone, the number of master junctions, tubules, branches, total length, and total tubule length in FMECs were significantly decreased (*P* < 0.001). It proved that the AS extract could promote angiogenesis, and this phenomenon could be reversed by LY294002.

### Effect of AS Extract on mRNA of PI3K/AKT Signaling Pathway in FMECs and GCs

**Figure 6:**
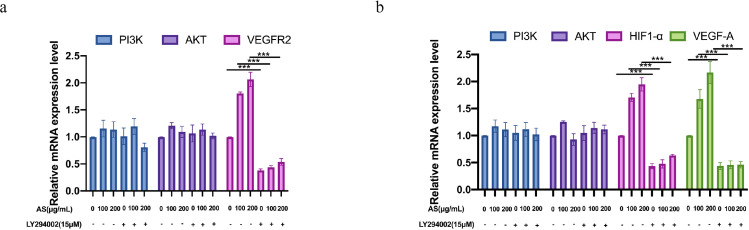
Effects of *Angelica sinensis* (AS) extract on mRNA expression in follicle microvascular endothelial-like cells of chicken (FMECs) and granulosa cells (GCs). (A) Relative mRNA expression of PI3K, AKT, and VEGFR2 in FMECs. (B) Relative mRNA expression of PI3K, AKT, HIF1-α, and VEGF-A in GCs. All experiments were performed in triplicate, and the data are the mean ± S.E.M (*P < 0.05, **P < 0.01, ***P < 0.001).

To explore the mechanism of AS extract enhancing angiogenesis, gene expressions of PI3K/AKT signaling pathway with and without LY294002 pre-treatment in FMECs and GCs were detected by RT-qPCR. AS extract caused a significant upregulation in VEGFR2 gene expression in FMECs (*P* < 0.001). In contrast, pretreatment of LY294002 obviously suppressed AS-induced VEGFR2 gene expression (*P* < 0.01; Figure 6A). AS extract caused a significant upregulation in HIF1-α and VEGF-A gene expression in GCs (*P* < 0.001). In contrast, pretreatment of LY294002 obviously suppressed AS extract-induced HIF1-α and VEGF-A gene expression (*P* < 0.01; Figure 6B). Furthermore, there were no statistic differences in PI3K and AKT gene expression after treatment of AS extract with and without LY294002 pretreatment in FMECs and GCs.

### Effect of AS Extract on VEGFR2-PI3K/AKT Signaling Pathway in FMECs

**Figure 7:**
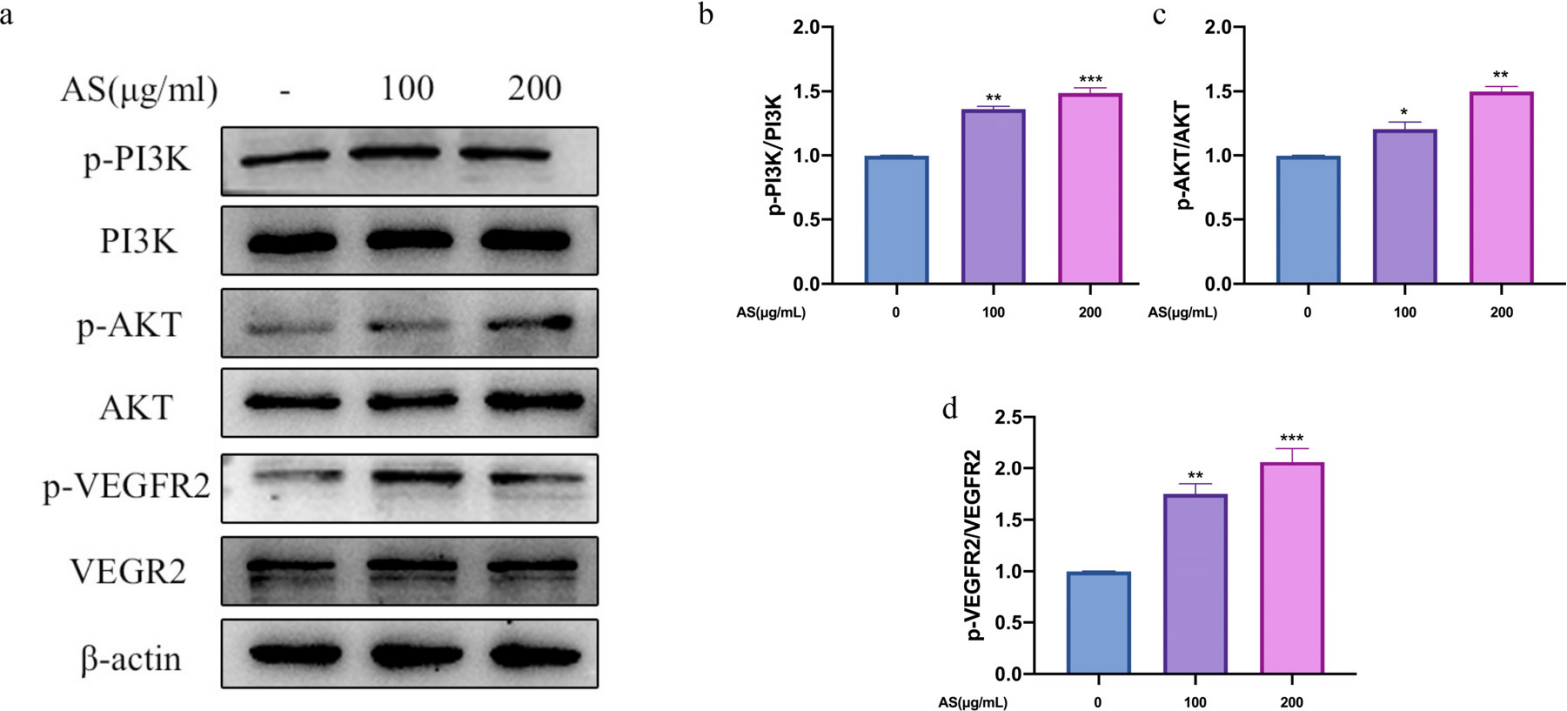
Effects of *Angelica sinensis* (AS) extract on phosphorylation of PI3K, AKT, and VEGFR2 in follicle microvascular endothelial-like cells of chicken (FMECs). β-actin was used as an internal reference protein. All experiments were performed in triplicate, and the data are the mean ± S.E.M (*P < 0.05, **P < 0.01, ***P < 0.001).

**Figure 8:**
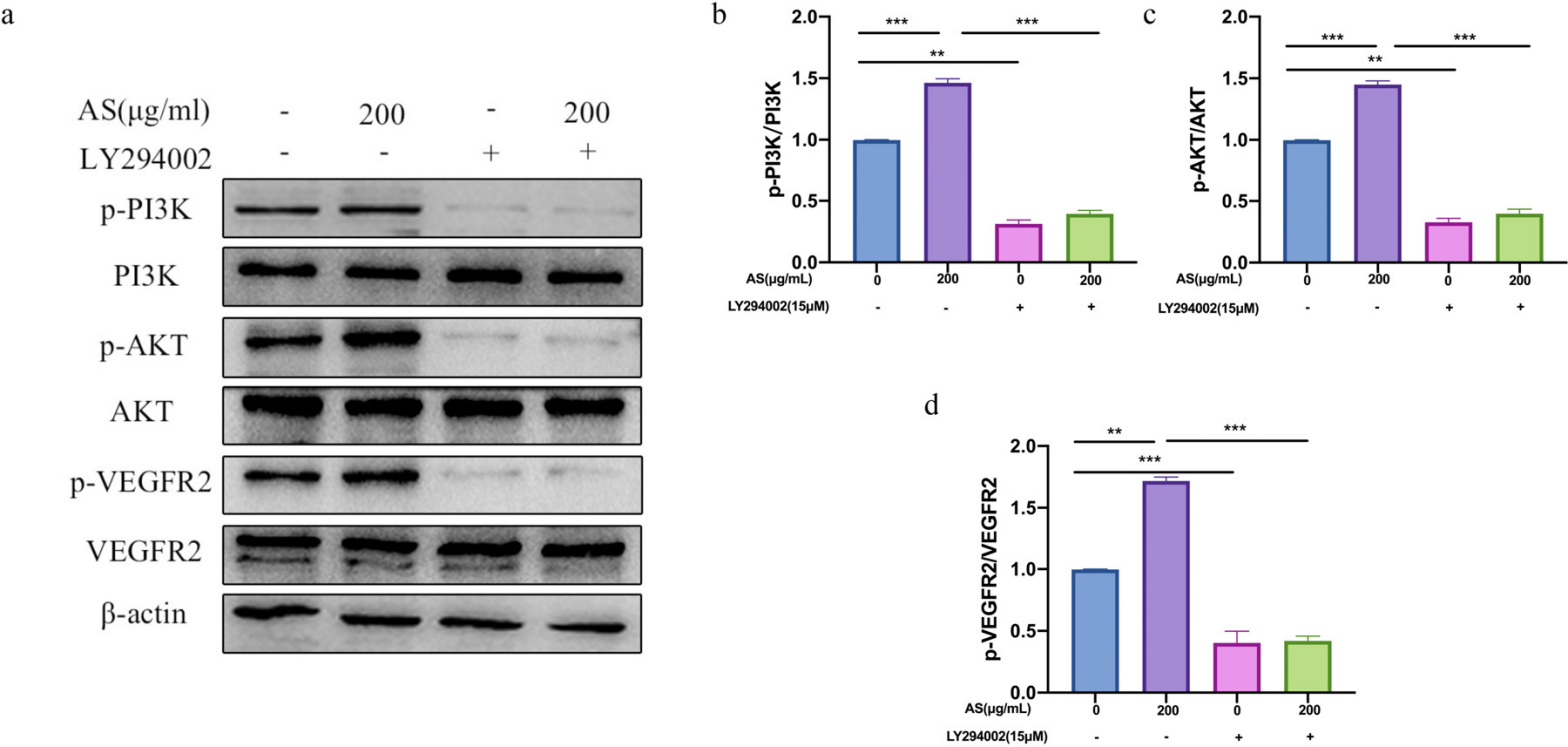
LY294002 (a PI3K inhibitor) inhibited the phosphorylation of PI3K, AKT, and VEGFR2 in follicle microvascular endothelial-like cells of chicken (FMECs) induced by *Angelica sinensis* (AS) extract. β-actin was used as an internal reference protein. All experiments were performed in triplicate, and the data are the mean ± S.E.M (*P < 0.05, **P < 0.01, ***P < 0.001).

In order to explore the mechanism of AS extract regulating angiogenesis, we analyzed the expression of proteins in the signaling pathway of angiogenesis by Western Blot. PI3K and AKT are key downstream proteins of VEGFR2, and activation of PI3K/AKT signaling pathway can promote endothelial cell growth, migration and angiogenesis (Martins et al., 2021). Compared with the control group, AS extract significantly increased the phosphorylation levels of VEGFR2, PI3K, and AKT in FMECs in a dose-dependent manner (*P* < 0.05, *P* < 0.01, *P* < 0.001; Figures 7A–7D). As shown in Figures 8A–8D, the phosphorylation levels of VEGFR2, PI3K, and AKT significantly reduced after treating with LY294002 alone (*P* < 0.01, *P* < 0.001). The phosphorylation levels of VEGFR2, PI3K and AKT did not increase significantly when treating with LY294002 and AS extract together. The above results indicated that AS extract promoted angiogenesis in theca layers by activating VEGFR2-PI3K /AKT signaling pathway in FMECs.

### Effect of AS Extract on PI3K/AKT-VEGF-A Signaling Pathway in GCs

**Figure 9:**
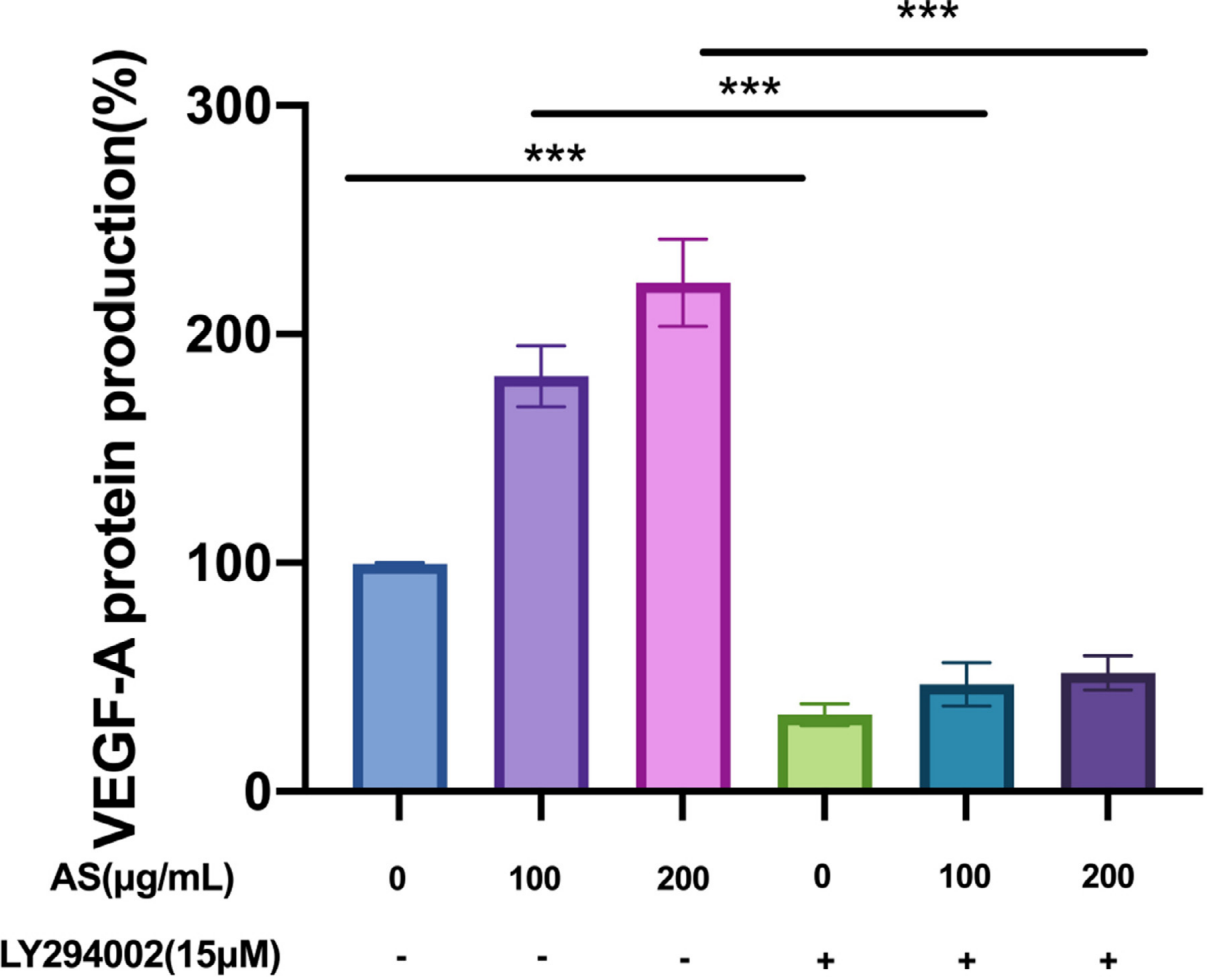
Effect of *Angelica sinensis* (AS) extract on granulosa cells (GCs) secreting VEGF-A. All experiments were performed in triplicate, and the data are the mean ± S.E.M (*P < 0.05, **P < 0.01, ***P < 0.001).

**Figure 10:**
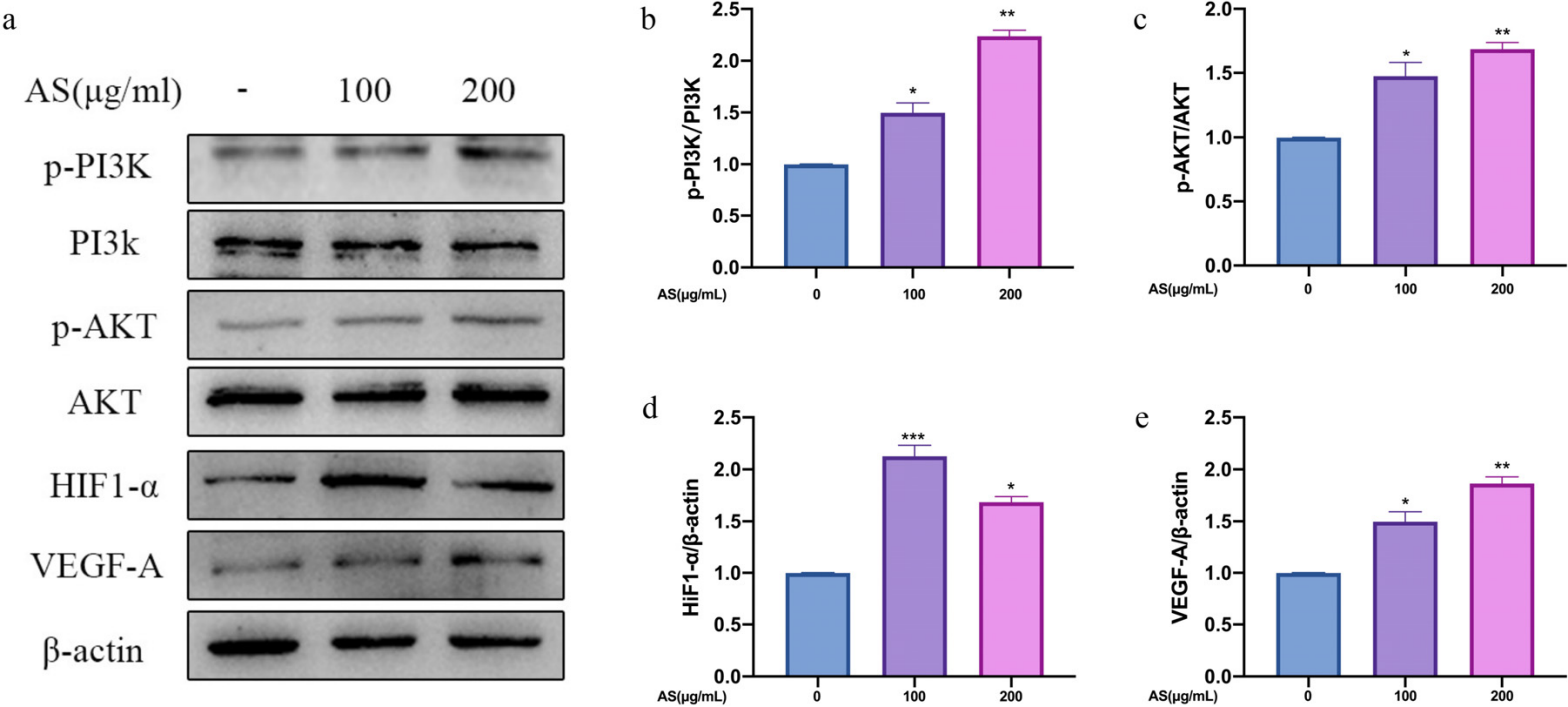
Effect of *Angelica sinensis* (AS) extract on PI3K/AKT-VEGF-A signaling axis in granulosa cells (GCs) (A–C) are phosphorylated PI3K and AKT proteins. (D, E) are HIF1-α and VEGF-A proteins. All experiments were performed in triplicate, and the data are the mean ± S.E.M (*P < 0.05, **P < 0.01, ***P < 0.001).

**Figure 11:**
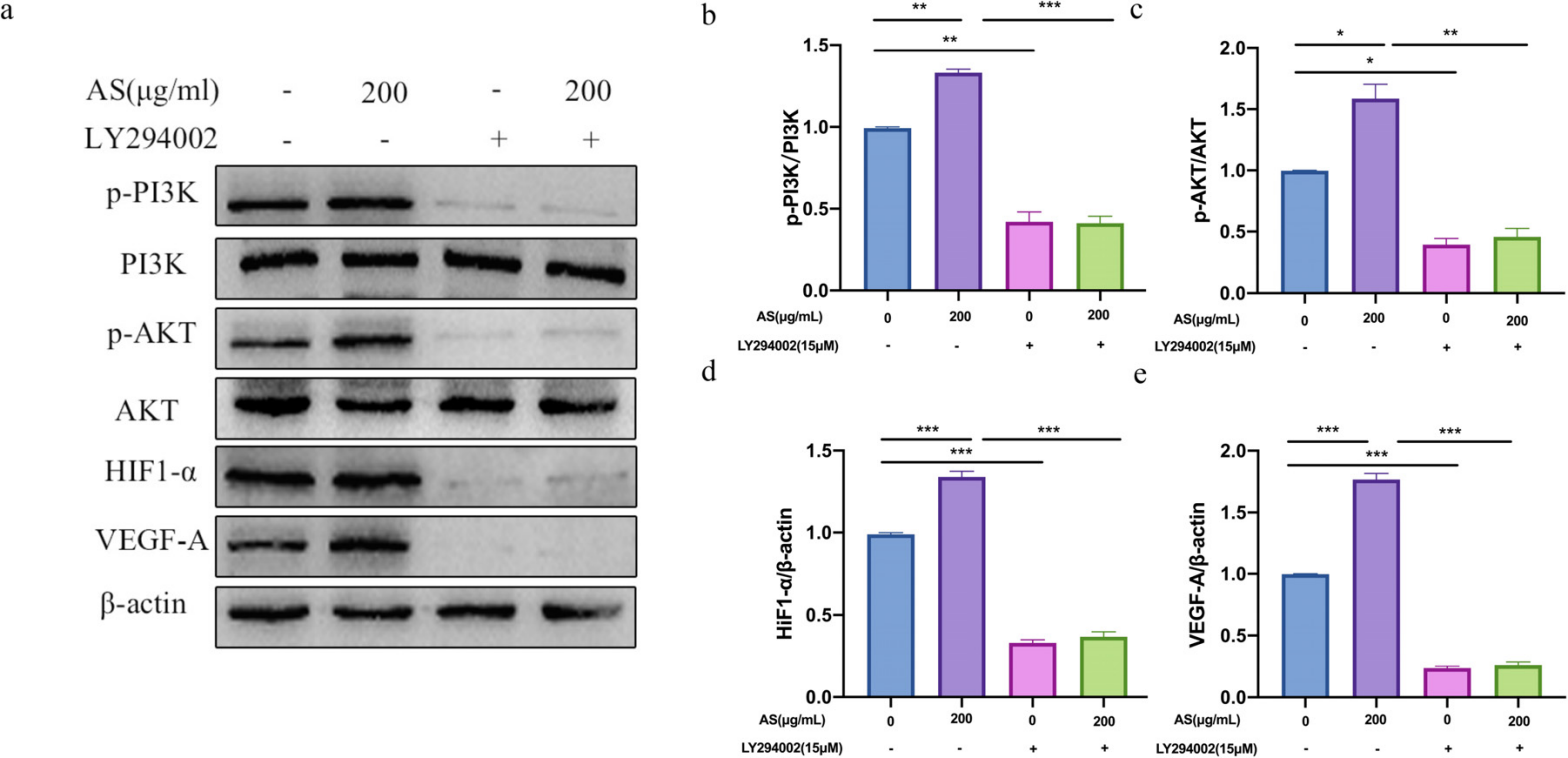
LY294002 (a PI3K inhibitor) inhibited the phosphorylation of PI3K and AKT proteins in granulosa cells (GCs) induced by *Angelica sinensis* (AS) extract. (A-C) are phosphorylated PI3K and AKT proteins. (D, E) are HIF1-α and VEGF-A proteins. All experiments were performed in triplicate, and the data are the mean ± S.E.M (*P < 0.05, **P < 0.01, ***P < 0.001).

VEGF-A secreted by granulosa cell layers can promote angiogenesis of theca layers. Thus, this experiment used ELISA to determine the secretion the cytokines of promotes angiogenesis. As show in Figure 9, compared with the control group, AS extract significantly increased the secretion of VEGF-A (*P* < 0.001). In contrast, pretreatment of LY294002 obviously suppressed the secretion of VEGF-A (*P* < 0.001). We detected the protein expression levels of PI3K/AKT-VEGF-A signaling pathway in GCs interfered with AS extract. Compared with the control group, AS extract significantly increased the phosphorylation levels of PI3K, AKT and upregulated VEGF-A, HIF1-α in GCs (*P* < 0.05, *P* < 0.01, *P* < 0.001; Figures 10A–10E). As shown in Figures 11A–11E, the phosphorylation levels of PI3K and AKT significantly reduced after treating with LY294002 (*P* < 0.05 *P* < 0.01), and the expression level of HIF1-α and VEGF-A significantly reduced (*P* < 0.001). Furthermore, the expression levels of p-PI3K, p-AKT, HIF1-α, and VEGF-A did not increase significantly when treating with LY294002 and AS extract together.

## DISCUSSION

The AS has been used as a traditional medicine for more than 1,000 yr in China, Korea, and Japan, and it is a common herbal and food supplement in China, Europe, and North America now (Han et al., 2021). A variety of food supplements have been proved to have angiogenic effects including *Abelmoschus Manihot* (Zhu et al., 2018) and Allicin (Shi et al., 2018). The AS contains a large number of active substances like ferulic acid and sodium ferulate, among which ferulic acid is one of the main pharmacological substances (Sung et al., 2014). Ferulic acid is a polyphenol compound that can regulate angiogenesis by promoting the expression of VEGF-A, PDGF, and HIF1-α (Cheng et al., 2017). There is evidence that AS extract enhances angiogenesis by activating phosphorylation of P-38 and JNK1/2, in addition to increasing angioppoietin-2 expression in brain damaged mice (Rong et al., 2013). Therefore, in this study, AS extract was used as experimental material to study the effects on angiogenesis of preovulatory follicles in chickens.

Eggs are one of the high-protein foods (Abeyrathne et al., 2013). Although the production of high yield commercial laying hens is high, the reproductive performance of commercial laying hens depends to a large extent on exogenous nutrient supply and follicular development. Good blood supply is the prerequisite to maintain high productivity and high egg quality of commercial laying hens. The rapid development of preovulatory follicles requires a large number of blood vessels to provide enough nutrition. Increasing the number of blood vessels in preovulatory follicles theca can promote the yolk deposition (Ma et al., 2020). Therefore, it is of great economic value to develop a safe and effective feed additive.

Both primordial and primary follicles receive sufficient nutrients and oxygen by passive diffusion from stromal blood vessels (Tamanini and De Ambrogi, 2004). The granulosa cell layer remains avascular until follicular developed, whereas the follicular theca layer is vascularized (Redmer and Reynolds, 1996). In the antral follicles of primates, follicles theca cells and GCs are always separated, and this anatomical structure is similar to preovulatory follicles of hens. It has been illustrated that blood vessels in the corpus luteum of primates initially develop from existing theca layer to granulosa cell layer because of VEGF-A secreted by granulosa cell layer (Fraser and Wulff, 2001). After the rapid formation of capillary in a short period of time, these new capillaries begin to connect with each other, forming a network of capillaries, thus obtaining a richer blood supply (Robinson et al., 2009). In this study, FMECs was extracted from developed preovulatory follicles of laying hens. The extract method is similar to separation of luteal microvascular endothelial cells (Sakurai et al., 2011), which is widely used for the separation of microvascular endothelial cells from heart, lung and brain. CD31 and vWF are recognized as specific markers of microvascular endothelial cells (Hou et al., 2020). The microvascular endothelial cells isolated and cultured in this study showed positive results of vWF and CD31 through immunofluorescence analysis. Matrigel is a solubilized basement membrane that mimics in vivo the environment for vascular endothelial cells culture applications. The cells we isolated could spontaneously form tube structures on Matrigel and proved to express more CD31 than the remaining cells by western blot analysis. In addition, preovulatory follicles of laying hens develop rapidly. The laying rate of hens in this experiment was more than 90%, which could ensure that the extracted follicles were preovulatory follicles under development. In summary, the cells we isolated were microvascular endothelial cells and suitable for studying angiogenesis during follicular development.

In order to better complete the cell to cell angiogenic response, VEGF-A binds to VEGFR on cell membranes (Gacche and Meshram, 2014). Previous studies have shown that VEGFR1, VEGFR2, and VEGFR3 have different genetic codes, but VEGFR1 and VEGFR2 have similar structures (Koch et al., 2011; Smith et al., 2015). VEGF-A mainly binds VEGFR1 and VEGFR2 to form blood vessels, while VEGF-C/D binds VEGFR3 to form lymphatic vessels (Simons et al., 2016). Although VEGFR1 has a higher affinity for VEGF-A than VEGFR2, but its kinase activity is lower. So VEGFR1 is considered to be A decoy receptor (Meyer et al., 2006). Therefore, most studies on angiogenesis focus on VEGFR2. This study found that AS extract could effectively improve the migration and invasion ability of FMECs. Our results showed that AS extract could effectively activate VEGFR-2, PI3K, and AKT, promote their phosphorylation, and promote angiogenesis of FMECs finally. Similar results have been obtained in recent studies that VEGF-A could activate the phosphorylation of VEGFR2, PI3K, and AKT (Zhu et al., 2018). A previous research (Yeh et al., 2011) showed that the volatile oil of AS exerted anti-angiogenic effects by inhibiting HUVEC proliferation, migration, and capillary-like tube formation on Matrigel. However, AS extract treatment exerted a protective effect on FMECs in our study. The reasons may be difference methods of preparation and different cells, in which Angelica sinensis extract plays different roles.

The avascular granulosa cell layer is located inside the vascularized follicular membrane. The angiogenesis of the theca layer is related to the angiogenic factors secreted by the granulosa cell layer (Robinson et al., 2009). Vascular endothelial cells need to absorb survival factors secreted from extracellular matrix (**ECM**) and surrounding cells, among which VEGF-A is an important substance for paracrine and endocrine nutrition support of endothelial cells (Campochiaro, 2015). GCs in follicles are the most important source of VEGF-A, and PI3K and AKT are vital upstream proteins of VEGF-A (Yang and Fortune, 2007). Thus, AS extract was added into the medium of GCs in this study. It was found that AS extract could activate phosphorylation of PI3K and AKT, and upregulate the expression of HIF1-α and VEGF-A. In the presence of HIF1-α, VEGF-A plays an important role in both physiological and pathological angiogenesis (Kelly et al., 2003).This conclusion is consistent with previous research (Meng et al., 2008).

PI3K inhibitor LY294002 was used to interfere with PI3K/AKT signaling pathway to further investigate the mechanism of AS extract promoting angiogenesis. In this experiment, the phosphorylation level of PI3K and AKT in cells and the migration and invasion ability of FMECs were inhibited by LY294002, which could increase by AS extract. The inhibit phenomenon was not improved when treated with LY294002 and AS extract together. It can be concluded that AS promoted angiogenesis of preovulatory follicles in chicken by activating the PI3K/AKT signaling pathway. However, in addition to PI3K/AKT signaling pathway, AS extract might has other target proteins for the role of follicle angiogenesis. These issues need further discussion.

**Figure 12:**
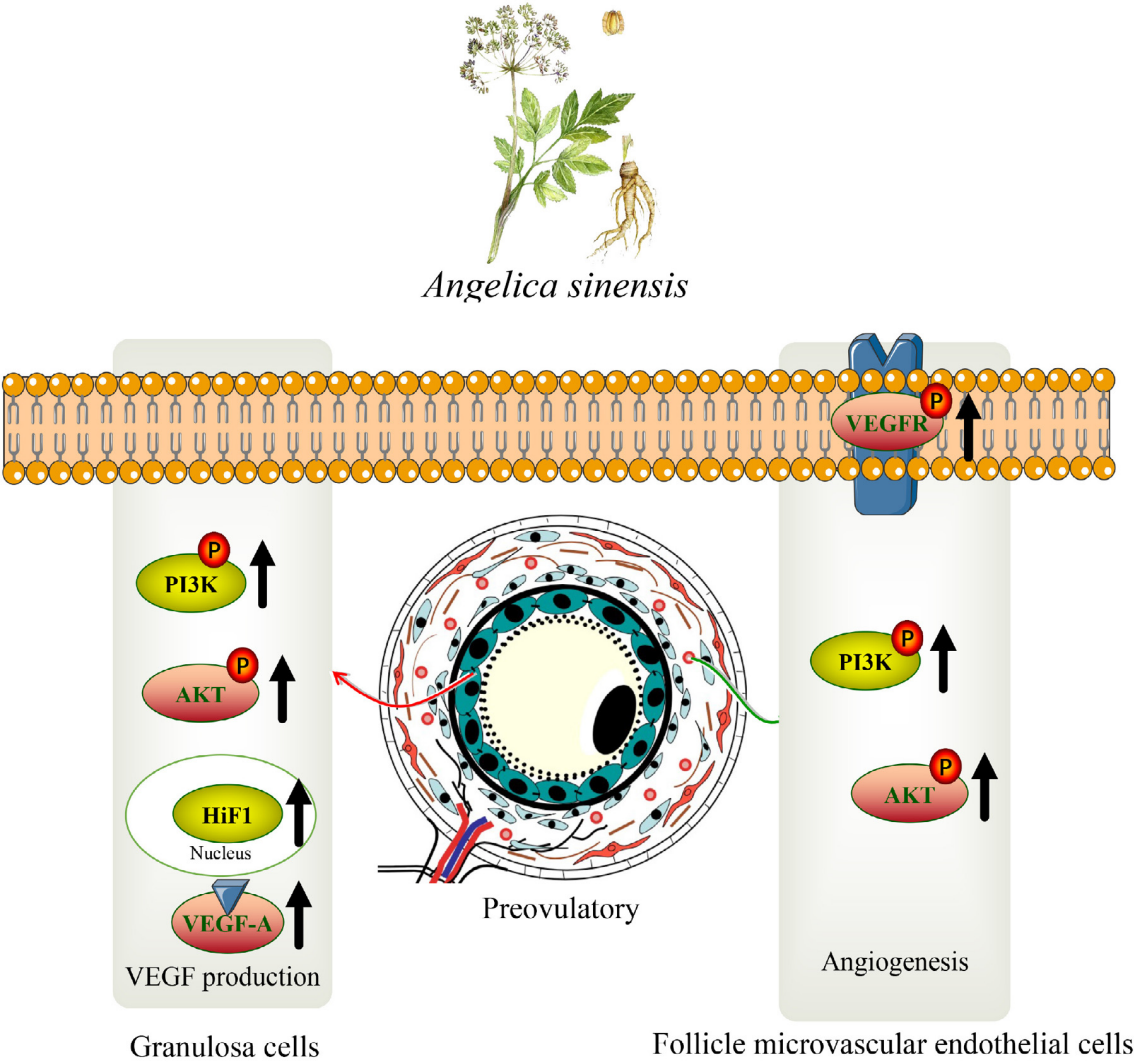
Main signaling pathways of *Angelica sinensis* (AS) extract promoting follicle microvascular endothelial-like cells of chicken (FMECs) invasion, migration and angiogenesis as well as promoting granulosa cells (GCs) proliferation and secretion of VEGF-A.

In conclusion, AS extract promotes the phosphorylation of VEGFR2 in FMECs by activating the PI3K/AKT signaling pathway, and enhances the invasion and migration ability of FMECs in vitro (Figure 12). In addition, AS extract can activate PI3K/AKT signaling pathway and upregulate the expressions of HIF1-α and VEGF-A in GCs. Therefore, we have reason to believe that AS extract can promote the follicle angiogenesis in hens in vitro.
